# Meeting Challenges in the Diagnosis and Treatment of Glaucoma

**DOI:** 10.3390/bioengineering12010006

**Published:** 2024-12-25

**Authors:** Karanjit S. Kooner, Dominic M. Choo, Priya Mekala

**Affiliations:** 1 Department of Ophthalmology, University of Texas Southwestern Medical Center, Dallas, TX 75390, USA; dominic.choo@utsouthwestern.edu (D.M.C.); priya.mekala@utsouthwestern.edu (P.M.); 2 Department of Ophthalmology, Veteran Affairs North Texas Health Care Medical Center, Dallas, TX 75216, USA

Glaucoma, a progressive and multifactorial optic neurodegenerative disease, still poses significant challenges in both diagnosis and management and remains a perpetual enigma. The disease’s insidious onset, combined with our reliance on subjective diagnostic tools, hinders early detection and timely intervention. Current treatments, while beneficial, often fail to provide consistent intraocular pressure (IOP) control, and management is further complicated by variability in disease progression, non-adherence to therapy, socioeconomic challenges, complications from surgical interventions, and new risk factors. For example, chronic stress has recently been implicated as a major risk factor in the pathogenesis of glaucoma [1,2]. The current Special Issue focusing on glaucoma has 14 excellent papers that discuss notable advancements aimed at addressing several challenges regarding this disease.

Several papers highlight the potential for the use of artificial intelligence (AI) in glaucoma care. Li-Han et al. developed a model that combines perimetry and optic coherence tomography (OCT) data to detect disease progression and achieved an F1 score of 0.60 within two years [3]. The model outperformed traditional methods like Bayesian regression (0.48), showcasing its potential for precise, early detection. Another novel model developed by Christoper et al. achieved an accuracy of 85% in predicting the need for glaucoma surgical interventions up to three years in advance. It utilized data such as patient demographics, medical history, clinical measurements, OCT, and perimetry [4]. Notably, the superiority of Vision Transformer (ViT) models, compared to convolutional neural networks (CNNs), in identifying glaucomatous optic neuropathy from fundus photographs was demonstrated by Hwang et al. [5].

The use of Ahmed valve shunts in Romania, as described by Barac et al., led to a reported 60% success rate in maintaining target IOP over five years, with a mean reduction to 17 mmHg [6]. Other innovations in filtering surgery are discussed by Ang et al. [7]. For example, limited deep sclerectomy-augmented trabeculectomy was shown to outperform conventional trabeculectomy, contributing to a mean IOP reduction from 29 ± 4.6 mmHg to 12.54 ± 1.67 mmHg at 12 months. A modified scleral tunnel technique dramatically reduced glaucoma drainage device (GDD) tube exposure rates to zero, as shown by follow-up examinations after 20 months. Similarly, for microinvasive glaucoma surgeries (MIGS), the introduction of the ab-externo approach for XEN stents improved IOP control and reduced postoperative interventions. Surprisingly, in the domain of cataract surgery, premium IOLs showed promising outcomes regarding spectacle independence and user satisfaction, specifically in patients with well-controlled, early-stage glaucoma or ocular hypertension [8]. However, their usage in advanced glaucoma must be considered with caution [8]. It was stressed that IOL selection should be individualized based on disease severity, visual expectations, and co-existing ocular conditions such as pseudo-exfoliation and ocular surface disease (OSD).

The importance of newer imaging technologies in predicting surgical outcomes was showcased by Tan et al. Using anterior segment OCT (AS-OCT), they found that successful blebs after deep sclerectomy had a significantly greater height (1.48 mm vs. 1.10 mm; *p* < 0.0001) and trabecular-Descemet window length (613.7 µm vs. 450.8 µm; *p* = 0.004) compared to failures [9]. Further contributing to the advancement of surgical outcomes, Fung et al. introduced a new high-throughput system that functions as a pre-animal testing platform for anti-fibrotic compounds [10]. Using this platform, they demonstrated that Verteporfin improves surgical outcomes by modulating the TGFβ-SMAD (Small Mothers against Decapentaplegic) pathway. In the same vein, Dave et al. reviewed emerging antifibrotic therapies, highlighting integrin inhibitors, and anti-LOXL2 antibodies as safer, more effective alternatives to traditional agents like mitomycin C [11].

Novel Rho-associated protein kinase (ROCK) inhibitors, particularly ZINC000000022706 and ZINC000034800307, with high binding affinities (−10.7 kcal/mol and −9.1 kcal/mol, respectively) were identified by Bodea et al. using bibliometric analysis and molecular docking [12]. A review of OSD by Kemer et al. demonstrated that 22–78% of patients on topical glaucoma medications experienced significant clinical side effects, and they encouraged the consideration of preservative-free formulations and adjunctive therapies like cyclosporine A [13]. Other novel emerging therapies, including extraocular and intraocular sustained-drug delivery systems, photobiomodulation, gene therapy, and stem cell applications, were reviewed as well.

Advancements in wearable technologies are also highlighted by Shean et al., with a focus on the Sensimed Triggerfish (24 h IOP measuring device) and drug-eluting contact lenses [14]. Notably, continuous monitoring using Triggerfish allowed for the identification of nocturnal IOP peaks as a critical risk factor for glaucoma, and drug-eluting contact lenses provided sustained IOP reductions of up to 30% over several weeks. Another review by Elhusseiny et al. emphasizes the predictive value of corneal hysteresis (CH), showing that a lower CH is associated with faster visual field deterioration (every 1 mmHg decrease in CH was associated with an additional 0.25% decline per year in visual field index) [15]. The potential to use Brillouin microscopy as a non-contact, three-dimensional assessment of corneal elasticity was also highlighted, as it offers a significant advancement compared to traditional methods that are solely reliant on IOP.

The interesting relationship between myopia and glaucoma is discussed by Vinod et al. [16]. The presence of myopia was shown to increase glaucoma risk due to structural vulnerabilities (thinning of the sclera and lamina cribrosa), with highly myopic eyes being 7.3 times more likely to develop glaucoma than emmetropic eyes. Overlapping features (optic nerve head changes and retinal nerve fiber layer thinning) may be overcome by utilizing the ever-expanding AI and advanced imaging technologies such as OCT-Angiography (OCTA) and Swept Source-OCTA.

Future advancements in glaucoma must continue to focus on early diagnosis and accurate prognostic evaluations, medical and surgical therapeutics, patient-centered technologies, and wearable theranostic devices (e.g., smart contact lenses) [14,17]. Emerging polymer-based long-term drug delivery systems have the potential for sustained IOP control and improved adherence. However, they still require continued optimization for biocompatibility and scalability to ensure their widespread application is possible [18,19]. Exciting improvements in surgical technique are on their way, such as the combination of traditional and minimally invasive approaches achieved by new advancements in GDDs and MIGS device designs [17,20,21]. Regarding AI tools, we have just begun to scratch the surface. These tools are being validated in diverse populations and their diagnostic and risk stratification capabilities continue to improve [22,23,24,25].

At the molecular level, neuroprotective strategies targeting oxidative stress-related mitochondrial dysfunction may offer promising pathways for retinal ganglion cell preservation [18,26,27,28,29,30]. Similarly, the impact of oxidative stress on trabecular meshwork cells and aqueous outflow resistance could also factor into targeted therapeutic approaches [31,32,33,34,35,36]. Epigenetic biomarkers (DNA methylation and histone modifications) and emerging gene therapies have the potential to revolutionize early diagnosis and therapeutic targeting [37,38,39,40,41,42]. Understanding the role of glial cell activation and its modulation of neuroinflammatory pathways may lead to improvements in the preservation of visual function and mitigation of neurodegeneration [19,43,44,45,46]. There is increasing focus on the relationship between gut microbiota and glaucoma, with emerging theories suggesting systemic roles for microbiome dysbiosis, as well as roles in neuroinflammation, including in autoimmune mechanisms [47,48,49,50,51,52,53,54,55]. Future research should prioritize understanding the mechanisms through which gut microbiota influence the development and progression of glaucoma.

Reviewing the submissions to this Special Issue on glaucoma, we have achieved our underlying aim of stimulating research and dialog among the global glaucoma research community. It is our firm belief that the future for glaucoma research looks exceptionally promising.
